# Tele-Operated Echography and Remote Guidance for Performing Tele-Echography on Geographically Isolated Patients

**DOI:** 10.3390/jcm5060058

**Published:** 2016-06-13

**Authors:** Philippe Arbeille, Kathryn Zuj, Arnaud Saccomandi, Elise Andre, Cedric De La Porte, Monica Georgescu

**Affiliations:** 1 Unite Medecine Physiologie Spatiale (UMPS-CERCOM) Faculte de Medecine, Tours 37032, France; Kathryn.zuj@gmail.com (K.Z.); georgescuemonica@yahoo.fr (M.G.); 2 MSP (Maison de Santé Pluridisciplinaire), Richelieu 37190, France; arnaud.saccomandi@gmail.com (A.S.); eliseandre2@gmail.com (E.A.); 3 MSP (Maison de Santé Pluridisciplinaire), Ligueil 37240, France; c.delaportedesvaux@wanadoo.fr

**Keywords:** tele-medicine, tele-echography, teleoperated probe, remote guidance

## Abstract

Objective: To evaluate the performance of three tele-echography systems for routine use in isolated medical centers. Methods: Three systems were used for deep (abdomen, pelvis, fetal) and superficial (muscle, thyroid, carotid artery) examinations: (a) a robotic arm (RA) holding an echographic probe; (b) an echograph with a motorized probe (MP); and (c) remote guidance (RG) where the patient site operator performed the examination assisted by an expert via videoconference. All systems were tested in the same medical center located 60 km away from the university hospital. Results: A total of 340 remote echography examinations were performed (41% RA and MP, 59% RG). MP and RA allowed full control of the probe orientation by the expert, and provided diagnoses in 97% of cases. The use of RG was sufficient for superficial vessel examinations and provided diagnoses in 98% of cases but was not suited for deep or superficial organs. Assessment of superficial organs was best accomplished using the MP. Discussion: Both teleoperated systems provided control of the probe orientation by the expert necessary for obtaining appropriate views of deep organs but the MP was much more ergonomic and easier to use than the RA. RG was appropriate for superficial vessels while the MP was better for superficial volumic organs.

## 1. Introduction

Echography is commonly used as the first imaging modality for medical diagnoses as it does not require large facilities and costly installations and is capable of providing rapid diagnoses in emergency situations. Access to ultrasound diagnostic systems can assist general practitioners (GPs) in making medical diagnoses, resulting in better medical assessments, faster decision-making, and quicker patient treatment. Therefore, there is a strong need to provide access to echography in insolated and remote areas potentially through the use of tele-echography.

Several methods of tele-echography, or remote echography, have been designed and validated for remote ultrasound examinations on isolated subjects and patients. These methods include the use of a robotic arm and motorized probe [1,2,3,4], remote image analysis or remote guidance through videoconferencing [5,6,7,8], and volume capture with three-dimensional (3D) reconstruction [9,10,11]. Each system has been developed for use in centers without trained sonographers. Additionally, as remote echography places a time demand on both the trained expert sonographer and patient site operator (potentially the attending GP), efforts have been made to ensure that remote examinations are performed within the timeframe of a normal medical consultation (less than 20 min). Despite most of these methods being technically and medically validated [1,2,3,6], there is still a need to determine which systems are appropriate for routine clinical use.

The following paper reports on the routine use of three different methods of tele-echography utilized in a single medical center. The systems were qualitatively assessed based on ease of use, diagnostic capabilities, and time required for examinations. It was hypothesized that one system would not be appropriate for all types of examinations in routine clinical practice and that each would provide benefits dependent on the type of examination to be performed.

## 2. Methods

Over a two-year period, three methods of tele-echography were utilized in a single medical center for clinical examinations. Patients were recruited for remote echography in the order that they arrived at the medical center and were not selected based on patient characteristics or pathology. Each patient was informed on the procedures and signed a consent form with the physician at the medical center. All protocols and procedures of the remote echography were approved by the University Hospital Ethics Committee (Tours, France).

Remote echography examinations, requested by the GP at the medical center, were scheduled based on the severity of the condition. Generally, examinations were performed within 24 h of the request, but some were also performed within 30 min in emergency situations. The examinations included both B-mode imaging and colour and PW Doppler assessments. For all examinations, a medical report was prepared by the expert sonographer and sent by e-mail to the attending physician within an hour after the completion of the examination.

**Figure 1:**
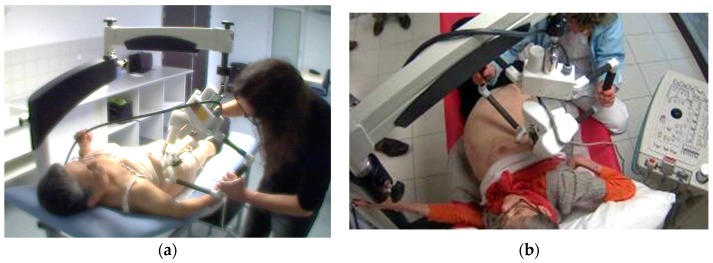
(**a**) Robotic arm (RA) suspended from its ground support and (**b**) located above a patient.

**Figure 2:**
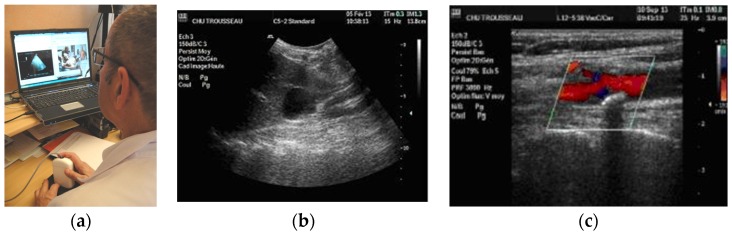
(**a**) RA expert center computer screen with video of the patient (left) and echographe screen (right); (**b**) Renal cyst image obtained using RA; (**c**) Carotid bifurcation with atheromatous plaque in color Doppler using RG.

**Figure 3:**
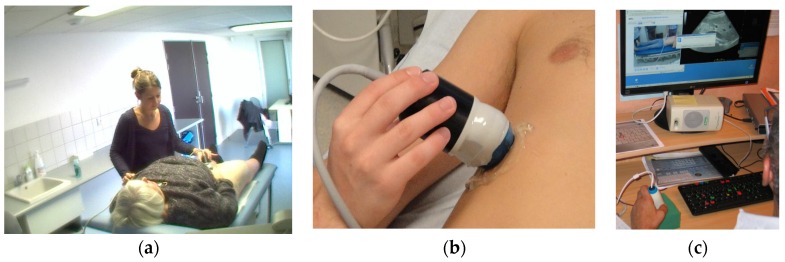
(**a**) General view of the isolated site with the MP held motionless on the patient by the non-sonographer operator (GP); (**b**) Probe with transducer tele-operated inside; (**c**) The expert with the dummy probe in hand, echograph function/settings teleoperation keyboard, and screen with ambient video and echographic view.

**Figure 4:**
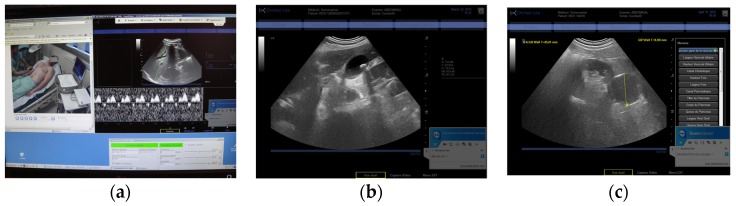
(**a**) Expert center computer screen for the MP with video of the patient (left), hepatic vein Doppler (right), and probe engine interface (bottom right); (**b**) View of gallbladder with lithiasis inside; (**c**) View of a renal cyst.

**Figure 5:**
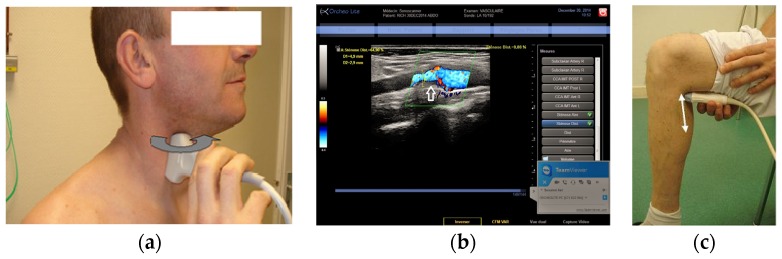
(**a**) Remote guidance (RG) for carotid artery; (**b**) View of a carotid stenosis in color Doppler by RG; (**c**) Location of the probe for calf vein visualization with RG.

The three tele-echography methods used included: (a) a teleoperated robotic arm (RA) holding an ultrasound probe; (b) a teleoperated echograph with a motorized probe system (MP); and (c) remote guidance (RG). The RA (Figure 1) system consisted of a large structure that held a standard ultrasound probe. From the expert center (Figure 2), a trained sonographer performed the tele-echography examination by manipulating a dummy probe which teleoperated the robotic arm [1,3]. Throughout the examination, the non-sonographer operator at the patient site was required to position the robotic arm over the patient and to adjust the echograph functions and settings as directed by the trained sonographer. The second system of tele-echography, MP, consisted of a modified commercial echograph with motorized probes [2]. The ultrasound probes used for this system contained internal motors to tilt and rotate the probe transducer which the trained sonographer teleoperated from the expert center (Figure 3 and Figure 4). In addition to controlling the probe orientation, the echograph used for this system was modified to allow for the teleoperation of settings and functions. The final system of remote echography evaluated utilized RG (Figure 5) [5,6,7]. For this system, the non-sonographer (GP) had the probe in hand and performed the echographic examination as directed by a trained sonographer via videoconference.

All the expert center devices were linked to the patient site through an Internet connection. The RA expert device was installed in a fixed location as it consisted of a personal computer an electrical module (25 cm × 20 cm × 15 cm, 1.5 kg) requiring an electrical power supply, and a dummy probe (150 g). Conversely, the MP and RG expert device were portable as they only consisted of a personal computer and a USB dummy probe (150 g) for the MP and a personal computer only for the RG systems.

Evaluation of the three methods of remote echography took place in two phases. During the first phase of the study (2014), the RA was used with a 3.5 MHz probe available for deep organ examinations and a linear 7.5 MHz probe for superficial structures. During the second phase of the study (2015), the MP system was introduced and the RA system removed. This system was capable of using a 3.5 MHz transducer for the deep organ examinations and a 10 MHz transducer for superficial structures. RG was used in both phases of the study with a linear 7.5 MHz probe being used in the first phase and a 17 MHz linear probe in the second phase. In addition, in the second phase of the study, RG examinations were performed using the teleoperated echograph. This echograph allowed the expert sonographer to teleoperate the echograph settings and functions, directly assisting the non-sonographer operator during the examination.

The choice of which system to use for the examination was made by the GP in the isolated site. At no time was the GP required to choose between the RA and MP as the RA was only available for the first phase of the study and the MP for the second phase after being developed in early 2015. Therefore, for all examinations the GP was required to choose between the teleoperated system available and RG.

Throughout the study, the number of patients assessed with each method was determined and GPs were questioned as to the reasons behind their choice of system. Additionally, at the completion of the study, GPs were asked to qualitatively assess the different systems based on the ability to provide a diagnosis and ease of use. As a quantitative assessment of system performance, the time required for examinations using each of the systems was compared using a Student’s *t*-test (Microsoft, Excel) with significance set at *p* < 0.05. Numerical values present the mean ± standard deviation.

## 3. Results

Over the two-year period, a total of 340 tele-echography examinations were performed. Examinations were most frequently requested for acute cholecystitis, renal cavity dilation, deep leg vein thrombosis, carotid vessel imaging in patients with cardiovascular risk factors (elevated blood pressure, recent infarcts, diabetes, high cholesterol, *etc.*), thyroid dysfunction, and muscular trauma. During the study period, examinations were requested, on average, one to two times per day, five days a week. Approximately 10% of remote echography examinations were requested for emergency situations.

A summary of the organs examined and the method used for each examination is presented in Table 1. Both the RA and the MP systems provided enough views of the deep organs of interest to deliver diagnoses in 97% of the cases (Figure 2 and Figure 4). For the superficial targets (organs and vessels), MP (Figure 4) and RG (Figure 5) provided the correct view of the organ necessary for the diagnosis in 98% of cases. Overall, tele-echography was not able to return enough information for a safe diagnosis in 11 of the 340 examinations (3.2%). These 11 cases involved imaging of deep organs (gall bladder, spleen, pancreas) on obese patients or patients with very poor echogenicity, and imaging of leg veins (femoral, tibial, gastrocnemius veins) on patients with substantial leg edema. For each of these cases, patients were referred to other medical centers for standard echography examinations.

The average examination time depended on the method used, the organ investigated, and patient characteristics (anatomy and echogenicity) with times ranging from 5 to 27 min. For deep organ examinations, remote echography with the MP system was significantly less (*p* = 0.012) than the time required with the RA system (Table 2). For superficial organs (blood vessels, thyroid, muscle), the teleoperation of the echograph settings and functions served to significantly reduce the time required for RG examinations (*p* = 0.017) compared to RG examinations with a conventional echograph (Table 2).

When asked to qualitatively assess the three remote echography systems, the isolated medical center GPs concluded that for deep organ assessments, the MP system was, for all cases, more ergonomic and easier to use. Additionally, they concluded that the MP was also the best method for investigating superficial organs. Finally, for the investigation of superficial vessels, RG was most appropriate.

## 4. Discussion

The current paper presents an assessment of three types of tele-echography used over a two-year period to perform 340 clinical examinations. Overall, each method of tele-echography presented similar diagnostic capabilities. However, the time required for examination and the ease of use differed between the various modalities. The results suggest that one system of tele-echography may not be the most appropriate for all ultrasound examinations commonly requested in clinical situations.

Each method of remote echography required an operator at the patient site (GP) to assist with the examination. Over the course of the two-year period, the non-sonographer operators became better at identifying the organ of interest displayed on the echograph, but were not more adept at acquiring the images requested for diagnosis. Therefore, the assessment of the different methods of tele-echography was dependent on the system performance and ease of use, and not the skill of the non-sonographer operator.

### 4.1. Deep Organ Examinations

Examination of the deep organs (such as the abdomen, pelvis, cardiac, fetal) required the use of either the RA or the MP system. These teleoperated systems allowed the expert sonographer to fine-tune the orientation of the probe to obtain the images required for diagnoses. In contrast, it was not possible to achieve the same degree of precision using only the verbal direction of the non-sonographer operator during RG; therefore, only the two teleoperated systems were appropriate for this type of examination.

Both teleoperated methods acted as an extension of the expert sonographer’s arm and hand, allowing the expert to tilt the probe (RA) or transducer (MP) ±55° from the vertical direction and to rotate the probe by ±180°. However, the MP was found to be more ergonomic and easier to move, allowing small translational movements (1–2 cm) and changes in the pressure of the probe against the patient’s skin, resulting in better image quality and reduced examination times.

The RA was designed in our laboratory in 1998 to facilitate remote echography for all types of examinations. As such, the arm was required to perform both angular motions (tilt, rotation). This resulted in a robot that was rather large and heavy (35 × 40 × 35 cm, 3.5 kg) and required a similarly large and heavy mechanical support structure (Figure 1). Although capable of obtaining images for diagnoses in a large number of situations, the non-sonographer operator at the patient site found the RA difficult to translate in the small increments necessary (<1 cm) to locate the proper acoustic window, resulting in longer examination times. In addition, the size and weight of the RA made it unsuitable for imaging more superficial structures and organs.

Advancements in ultrasound probe technology lead to the recent development of the teleoperated motorized probe system. Much smaller and lighter than the RA (430 cm^3^, 400 g), the non-sonographer operators (GPs) found the MP system much easier to maneuver which resulted in shorter examination times. In addition, the low weight and general maneuverability of the motorized probes allowed for imaging along the side, and under the costal border of the patient, providing access to different acoustic windows without requiring the patient to change body position. As the MP system is limited by the availability of the specifically designed motorized probes, the probes constructed for the MP system were multi-frequency (3–5 MHz, and 5–15 MHz) to increase functionality. In contrast, the RA system could be modified to use a wide range of conventional ultrasound probes. However, due to its complex robotic technology, the RA system is much larger and more expensive than the MP system (50% higher cost). In the current study period, the medical professionals at the patient site and the trained sonographer did not have the option to choose between the RA and the MP systems as they were not available during the same time periods. However, after using each system for a minimum of six months, both medical professionals and patients expressed a preference for the MP method.

### 4.2. Superficial Organ Examinations

Similar to deep organ examinations, the MP system was preferred for examinations of superficial organs. Once again, the RA was found to be too difficult to locate accurately over the acoustic window and follow the curvature of an organ, due to the size and weight of the system. In contrast, the small motorized probe was easy to position and hold throughout the examination and did not applied to much pressure on the organ. Once in place, the MP system allowed the expert to entirely investigate the inside of the organ of interest by teleoperating the transducer orientation. Additionally, using this MP system, the expert could scan the entire volume of the organ, store the resulting video, and reconstruct a 3D image for later assessment at the expert center [9].

As with deep organ assessments, RG was not suited to the examination of superficial organs. The non-sonographer operators at the patient site were not capable of performing the fine probe adjustments necessary to obtain the quality of images in different planes necessary for diagnoses. It should be noted that to perform echography examinations, sonographers require extensive training over a six-month period for each organ group to be assessed, imaging approximately 8 h per day. Thus, only an expert sonographer is capable of quickly scanning the inside of the organs for diagnoses. However, with training, the non-sonographer operator was able to obtain images for assessments of superficial vessels which are easier targets.

### 4.3. Superficial Vessel Examinations

In both phases of the study, RG was found to be the most appropriate for assessments of superficial blood vessels. In contrast to the other methods of tele-echography assessed, RG did require training of the non-sonographer operator. However, it was noted over the course of this investigation that, with one week of training, the non-sonographer operator could become sufficiently proficient at obtaining long and short axis views of superficial vessels. It is very easy for any operator, even a non-sonographer, to visualize a cross-sectional view (black circle in the image) of a superficial artery or vein. With training, the operator was then able to rotate the probe 90 degrees to turn the transverse image of a dark beating circle into the long rectangular black area of a longitudinal image. Under the direction of the expert sonographer, the non-sonographer operator then translated the probe to the appropriate position (e.g., carotid bifurcation) and adjusted the probe to obtain a clear longitudinal image for diagnosis.

During the first phase of the study, the 7 MHz probe was available for use with the RA and RG. In this case, RG was chosen as the most appropriate method due to the size and weight limitations of the RA and the ability of RG to provide images for diagnoses. In the second phase, a higher frequency 17 MHz two-dimensional (2D) probe was available for RG examinations where only a 10 MHz probe was available for the MP system. In this case, RG was again chosen as the most appropriate method for superficial vessel examinations as the higher frequency of the transducer provided images of better quality for diagnoses. Additionally, the higher frequency probe (like every conventional probe) used with RG has a narrow head which allows the operator to reduce the distance between the vessels of interest and the probe transducer by applying slight pressure, thereby improving image quality. In contrast, the head of the motorized probe is much wider and does not allow for a similar maneuver. Thus, it was agreed that for superficial vessels (carotid artery, leg artery and veins), priority should be placed on the echographic performance of the probe (high frequency, narrow foot print) rather than teleoperation ability.

During the second phase of the study, the ability of the expert sonographer to teleoperate the echograph at the patient site proved to be a real advantage. With RG the non-sonographer operator is generally required to control all the echograph functions and settings under the verbal direction of the expert sonographer. This requires additional training and skill of the operator to maintain the proper image (fix the hand orientation) while making adjustments on the echograph. The introduction of the teleoperated echograph in the second phase of the study allowed the non-sonographer operator to focus on maintaining the proper image (hand motionless) while the expert controlled the echograph functions and settings (for example. Doppler, gain, video and image saving, measurements). This resulted in more efficient examinations with shorter examination times.

For all types of examinations, the ability to move the MP and RG expert center devices (PC and dummy probe) was considered by the experts to be a major advantage. With these systems the MP and RG expert devices could be activated from any place simply with Internet access (medical centers, home, overseas hotels, vehicles with 3G mobile phone) and did not require travel to the fixed RA control location. Therefore, these systems provided greater flexibility for the expert sonographers while on call or simply not present in the hospital.

## 5. Conclusions

Presently, the medical reliability and usefulness of remote echography for isolated patients have been demonstrated, but it appears that no one single method is appropriate for all types of exams requested in routine clinical practice. The use of the MP system provided a more ergonomic means of remote echography that was suited for a wide range of examinations including both superficial and deep organs. However, it was found that for superficial blood vessels, RG was just as efficient and provided better quality images. This study also showed that the ability of the expert sonographer to teleoperate the settings and functions of the echograph during RG examinations improved the quality of the images and reduced the time needed for the examination. Of the three methods reviewed in this paper, each presented similar diagnostic capabilities, but differed in suitability for the different organ categories investigated and for the isolated medical center environment and activity.

## Figures and Tables

**Table 1:** Number and type of examinations performed with each method of remote echography.

	Type of Remote Echography Used				
Type of Examination	Robotic Arm	Motorized Probe	Remote Guidance Conventional Echograph	Remote Guidance Teleoperated Echograph	Total Number of Patients
Abdominal Organs	37	52	--	--	**89**
Kidney	7	10	--	--	**17**
Pelvis	3	3	--	--	**6**
Neck Blood Vessels	--	--	69	47	**116**
Leg Veins	--	--	28	28	**56**
Thyroid	--	14	13	--	**27**
Muscle	--	13	3	13	**29**
Total Number of Patients	**47**	**92**	**113**	**88**	**340**

Values show the number of examinations performed for each of the tele-echography systems investigated. Over the course of the two-year period, 340 examinations were performed with the RA and MP systems used for deep organ examinations and RG was used for superficial blood vessel examinations.

**Table 2:** Duration of examination for each category of organ and each method of remote echography.

	Time Duration for Each Type of Remote Echography Used				
Type of Examination	Robotic Arm	Motorized Probe	Remote Guidance Conventional Echograph	Remote Guidance Remote Echograph	*P* Value
Deep Organs	14.1 ± 5.3	11.5 ± 4.3 *	--	--	(*p* = 0.012)
Superficial Organs	--	--	11.3 ± 4.3	9.6 ± 3.9 *	(*p* = 0.017)

Values (mean ± SD) show the average time of examination for each of the methods of remote echography used. Deep organ examinations with the MP were significantly shorter than with the RA (*p* = 0.012). Similarly, examination times for RG with a teleoperated echograph were significantly shorter than using a conventional echograph (* *p* = 0.017).
